# ^18^F-florbetaben Aβ imaging in mild cognitive impairment

**DOI:** 10.1186/alzrt158

**Published:** 2013-01-16

**Authors:** Kevin Ong, Victor L Villemagne, Alex Bahar-Fuchs, Fiona Lamb, Gaël Chételat, Parnesh Raniga, Rachel S Mulligan, Olivier Salvado, Barbara Putz, Katrin Roth, Colin L Masters, Cornelia B Reininger, Christopher C Rowe

**Affiliations:** 1 Department of Nuclear Medicine and Centre for PET, Austin Health, 145 Studley Road, Heidelberg, VIC 3084, Australia; 2 Department of Medicine, University of Melbourne, Parkville, VIC 3010, Australia; 3 Mental Health Research Institute, 155 Oak Street, Parkville, VIC 3052, Australia; 4 Centre for Research on Aging, Health, and Wellbeing, 63 Eggleston Road, The Australian National University, Acton, ACT 2600, Australia; 5 CSIRO Preventative Health National Research Flagship, The Australian e-Health Research Centre - BioMedIA, Herston, QLD 4029, Australia; 6 Bayer Pharma AG, Müllerstraße 178, 13353 Berlin, Germany

## Abstract

**Introduction:**

^18^F-florbetaben and positron emission tomography were used to examine the relationships between β-amyloid (Aβ) deposition, cognition, hippocampal volume, and white matter hyperintensities in mild cognitive impairment (MCI).

**Methods:**

Forty-five MCI participants were evaluated. A neocortical standardized uptake value ratio threshold ≥ 1.45 was used to discriminate high from low Aβ burden. Correlations were adjusted for age, gender and years of education.

**Results:**

High Aβ burden was found in 53% of MCI. Regression analyses showed standardized uptake value ratio (*r *= -0.51, *P *= 0.0015) and hippocampal volume (*r *= 0.60, *P *= 0.024) both contributing to episodic memory impairment in independent fashion. White matter hyperintensities correlated with nonmemory cognition, and this correlation was particularly associated with Aβ burden.

**Conclusion:**

Higher Aβ deposition in MCI is associated with more severe memory impairment and is contributing to early amnestic symptoms independent of hippocampal atrophy.

## Introduction

The leading etiological hypothesis of Alzheimer's disease (AD) points to excessive brain β-amyloid (Aβ) that aggregates to form extracellular plaques and vascular wall deposits [1]. With increasing prevalence and associated cost of care and the likelihood of greater benefit if therapies are applied early, earlier and more accurate identification of AD has become a research priority.

Dementia is usually preceded by a transition period of cognitive decline commonly referred to as mild cognitive impairment (MCI). Characterized by an objective impairment of memory and/or other cognitive domains, MCI is not severe enough to significantly interfere with activities of daily living [2]. The prevalence of MCI in people aged 65 is believed to be 10 to 20%, with over 10% who have been classified as MCI converting to dementia per year [3]. Histopathologic studies on brains of MCI subjects have shown characteristic AD pathology including Aβ plaques and neurofibillary tangles in the majority of cases [4]. MCI has been further classified based on whether memory has been affected (amnestic MCI) or spared (nonamnestic MCI), and whether the cognitive deficit affected is mainly in one cognitive domain (single-domain MCI) or more than one domain (multidomain MCI). Hence, MCI can be classified into four clinical subtypes: nonamnestic single-domain, nonamnestic multiple domains, amnestic single-domain (asMCI), and amnestic multiple domains (amMCI). These subtypes probably differ in etiology and outcome. Impaired episodic memory, which characterizes asMCI and amMCI, is thought to be a prodromal condition for AD [3,4].

The new research diagnostic criteria for AD and MCI allow for Aβ imaging in the workup of individuals with cognitive impairment [5,6]. Non-invasive Aβ imaging to confirm the presence of AD neuropathology could aid in early differential diagnosis, identify at-risk individuals, help predict or monitor disease progression, and potentially evaluate the response to disease-specific therapy. ^11^C-Pittsburgh Compound B (PiB) has been the most widely used agent in dementia research to assess Aβ burden *in vivo *[7]. The major disadvantage of PiB is that it is radiolabeled with carbon-11, which has a short decay half-life (20 minutes) that limits its use to centers with an onsite cyclotron and ^11^C-radiochemistry expertise.

To overcome these limitations, a number of novel fluorine-18 Aβ imaging tracers such as ^18^F-florbetaben (BAY 94-9172) [8-10], ^18^F-florbetapir (AV45) [11,12] and ^18^F-flutemetamol (GE067) [13,14] have been developed. The 110-minute radioactive decay half-life of fluorine-18 allows centralized synthesis and regional distribution of these tracers as currently practiced worldwide in the supply of ^18^F-fluorodeoxyglucose for routine clinical positron emission tomography (PET) imaging.

^18^F-florbetaben (FBB; trans-4-(*N*-methyl-amino)-4"(2-(2-(2-[^18^F] fluoro-ethoxy)ethoxy)-ethoxy)stilbene), developed by Avid Radiopharmaceuticals (Philadelphia, USA) and Bayer-Schering Pharma (Berlin, Germany), has been shown to bind with high affinity to Aβ in brain homogenates and selectively labeled Aβ plaques and cerebral amyloid angiopathy (CAA) in AD tissue sections [15]. After injection into Tg2576 transgenic mice, *ex vivo *brain sections showed localization of FBB in regions with Aβ plaques as confirmed by thioflavin binding [16]. At the tracer concentrations achieved during human PET studies, FBB did not show binding to α-synuclein in Lewy bodies or to tau lesions in postmortem cortices from dementia with Lewy bodies, AD or frontotemporal lobar degeneration patients [17]. In human studies, cortical retention of FBB was significantly higher in AD patients compared with age-matched controls and frontotemporal lobar degeneration patients, with binding matching the reported postmortem distribution of Aβ plaques [9]. Phase II clinical studies further confirmed these results [8]. FBB is highly correlated with ^11^C-PiB (*r *= 0.97 with a slope of 0.71) [18], and was used to detect the presence or absence of AD pathology in the brain in participants with a wide spectrum of neurodegenerative diseases including a few MCI participants [10]. Phase III studies for FBB have reached completion [19].

Human postmortem studies have shown that while soluble Aβ oligomers and the density of neurofibrillary tangles strongly correlate with neurodegeneration and cognitive deficits, the density of Aβ insoluble plaques does not [20-24] and Αβ burden as assessed by PET does not strongly correlate with cognitive impairment in AD patients [25,26]. The severity of tau pathology in AD patients is closely related to neuronal loss [27], hippocampal atrophy [28,29] and memory impairment [30,31]. Amyloid imaging studies in MCI have shown an association between Aβ burden and memory [32], an association that is believed to be mediated by hippocampal atrophy [33]. Vascular pathology, as reflected in white matter hyperintensities (WMH), has been shown to be associated with cognitive impairment, particularly affecting working memory and executive function, as well as visuospatial abilities among people with MCI [34].

The purpose of this study was to characterize FBB binding in a well-characterized MCI cohort, and to explore the relationships of Aβ burden cognitive performance, hippocampal volume (HV), and WMH.

## Materials and methods

### Participants

Forty-five participants fulfilling Petersen's criteria for MCI [3] were recruited between June 2008 and December 2009 from memory disorder specialists. Fifteen healthy older controls and 15 patients who met National Institute of Neurological Disorders and Stroke-Alzheimer's Disease and Related Disorders Association criteria for probable AD, which were previously described in an earlier study [9], were used for comparison against the MCI cohort.

Consistent with the consensus criteria for MCI at the time of enrolment [3], all participants (and their next of kin) reported a history of cognitive decline and had objective cognitive impairment on neuropsychological assessment but remained generally independent in daily activities. In addition, participants had to be at least 60 years of age, had at least 7 years of formal education, spoke fluent English, were capable of giving informed consent, had a reliable informant capable of giving a collateral history, were able to tolerate a brain magnetic resonance imaging (MRI) scan, did not meet the National Institute of Neurological Disorders and Stroke-Association Internationale pour la Recherché et l'Enseignement en Neurosciences criteria for the diagnosis of vascular dementia, and scored ≥ 24 on the Mini-Mental State Examination (for detailed exclusion criteria, see Table S1 in Additional file 1). These participants were referred from local specialist public and private memory disorders clinics upon being diagnosed with MCI and had no other evidence of significant neurodegenerative disease, moderate or severe psychiatric illness, drug or alcohol dependence, or participated in any anti-Aβ therapeutic trial prior to enrolment.

The recruitment criterion was defined as having at least one test score falling 1.5 standard deviations below published means. For precision, subsequent classification of participants into MCI subtypes by Petersen's criteria [3] was based instead on test scores falling 1.5 standard deviations below the mean of a carefully screened and demographically well-matched cohort living in the same region as the participants. This cohort consisted of 45 healthy older participants from the Australian Imaging Biomarkers and Lifestyle flagship study of ageing [35] with no history of cognitive decline who had negative brain PiB scans, normal brain MRI, Clinical Dementia Rating = 0 and Clinical Dementia Rating sum of boxes = 0, and had no psychiatric illness.

Approval for the study was obtained from the Austin Health Human Research Ethics Committee. Written informed consent for participation was obtained from all participants prior to screening. Safety monitoring consisted of clinical observation, baseline ECG, hematology and biochemistry testing and measurement of vital signs before and after tracer injection. Vital signs, hematology, and biochemistry testing were repeated 1 week after injection. Participants were asked about possible adverse events after their PET scan and 1 week after injection.

### Neuropsychological evaluation

Neuropsychology evaluation was conducted within 24.5 ± 15.5 days of the FBB PET scan by a licensed neuropsychologist. Evaluation consisted of the Mini-Mental State Examination, the Clinical Dementia Rating, the California Verbal Learning Test Second Edition, the Rey Complex Figure Test (RCFT), Logical Memory I and II (Wechsler Memory Scale; Story A only), the Controlled Oral Word Association Test, Categorical Fluency, the Boston Naming Task (30-item version), Digit Symbol-coding and Digit Span.

Individual composite episodic memory *z *scores (EM) were generated in 44 participants by averaging the *z *scores for delayed recall trials of the RCFT, the California Verbal Learning Test Second Edition, and Logical Memory II. The RCFT delayed recall score was missing for another participant and was substituted with the RCFT immediate delay score because the relationship between scores on the immediate and the delayed recall trials was very strong (*r *= 0.93). Composite nonmemory *z *scores in all 45 participants were calculated by averaging the *z *scores for the Boston Naming Task, the Controlled Oral Word Association Test, Categorical Fluency, Digit Span, Digit Symbol-coding and RCFT copy [32].

### Image acquisition

#### Magnetic resonance imaging

A three-dimensional T1-weighted magnetization prepared rapid gradient echo sequence and a fluid-attenuated inversion recovery sequence were performed on either a 1.5 T or a 3 T magnetic resonance scanner prior to the PET scan.

#### ^18^F-florbetaben imaging

Labeling was carried out in the Austin Health Centre for PET, as previously described [9]. Mean specific activity at the time of injection for MCI was 60 ± 29 GBq/μmol. Imaging was performed with a three-dimensional GSO Philips Allegro PET camera. A 2-minute transmission scan using a rotating ^137^Cs source was performed for attenuation correction immediately prior to scanning. Each MCI participant received on average 286 ± 19 MBq FBB intravenously over 38 ± 17 seconds. Images were reconstructed using a three-dimensional RAMLA algorithm (Philips, Cleveland, USA). Images obtained between 90 and 110 minutes post injection were used for the analysis.

### Image analysis

All image analysis was performed by experienced operators blind to the clinical status and cognitive test scores of the subjects.

Extraction of HVs from the three-dimensional magnetization prepared rapid gradient echo MRI data in 43 MCI cases was performed using a commercial, US Food and Drug Administration-approved, fully automated volumetric measurement program (NeuroQuant^®^) [36]. Preprocessing of the fluid-attenuated inversion recovery images was performed to correct for bias field effects and remove noise using anisotropic diffusion prior to manual segmentation of deep WMH. Manual segmentation of the WMH (PR) was performed using MRIcro software [37]. The total WMH volume in each MCI subject was calculated, as well as the number of individual lesions. All volumes were normalized for head size using the total intracranial volume, defined as the sum of gray matter, white matter and cerebrospinal fluid volumes.

Spatial normalization and co-registration of the PET and MRI images was performed using SPM8 [38]. PET images were processed with a semiautomatic volume of interest method. This method used a preset template of narrow cortical volume of interest that was either applied to the spatially normalized MRI and then transferred to the co-registered FBB scan or applied directly to the spatially normalized FBB scan. Minor manual adjustments were made to ensure that overlap with white matter and cerebrospinal fluid was minimized. Mean radioactivity values were obtained from the volume of interest for the cortical, subcortical and cerebellar regions. The cerebellar cortical volume of interest was placed taking care to avoid cerebellar white matter. All volume of interest placement was performed by a single experienced operator (VLV) blind to the clinical status of the individuals. No correction for partial volume effects was applied to the PET data.

The standardized uptake value, defined as the decay-corrected brain radioactivity concentration normalized for injected dose and body weight, was calculated for all regions. These values were then used to derive the standardized uptake value ratio (SUVR), which was referenced to the cerebellar cortex. Neocortical Aβ deposition was expressed as the average SUVR of the mean for the following cortical regions of interest: frontal (consisting of dorsolateral prefrontal, ventrolateral prefrontal, and orbitofrontal regions), superior parietal, lateral temporal, lateral occipital, and anterior and posterior cingulate.

To identify a SUVR cutoff point, a hierarchical cluster analysis of the neocortical SUVR of FBB scans in healthy control participants was performed similar to that previously described [10]. The cutoff value for high neocortical SUVR in this study was defined as ≥ 1.45.

### Statistical analysis

Independent-sample *t*-tests were used to compare means of MCI subtypes with healthy controls and AD patients, and to compare means within the MCI subtypes. Categorical differences were assessed using Fisher's exact test. Pearson's or Spearman's rank correlation analyses were conducted to assess the degree of linear relationship between neuroimaging variables (SUVR, HV, WMH) with composite EM and nonmemory *z *scores, adjusting for age, gender and years of education. Data are presented as mean ± standard deviation unless otherwise stated. Adjustment for multiple testing was not performed.

### Role of the funding source

The funding sources had no role in the data analyses and interpretation. The corresponding author had full access to all data presented in this study and had final responsibility for the decision to submit for publication.

## Results

### Population characteristics

Table 1 summarizes the demographic characteristics of the 45 MCI participants subclassified by Petersen criteria [3]. The table also details the demographic characteristics of the previously reported healthy controls and AD subjects for comparison [9]. Twenty-nine participants were classified as having amMCI and 12 were assessed as having asMCI. Two participants were classified as nonamnestic single-domain MCI and two as nonamnestic multiple-domain MCI. Given the low number of nonamnestic MCI cases, they were grouped together for all analyses.

**Table 1:** Demographics

				MCI subtype		
				
	HC (*n *= 15)	AD (*n *= 15)	All MCI (*n *= 45)	naMCI (*n *= 4)	asMCI (*n *= 12)	amMCI (*n *= 29)
Age	68.8 ± 6.5	69.5 ± 9.7	72.7 ± 6.5	74.5 ± 6.8	70.2 ± 6.9	73.5 ± 6.3
Years of education	13.3 ± 3.6	12.8 ± 3.9	13.6 ± 3.6	14.8 ± 1.7	15.2 ± 3.9	12.8 ± 3.6
Males, *n *(%)	8 (53)	10 (66.7)	29 (64)	4 (100)	7 (58)	18 (62)
MMSE	29.5 ± 0.8	23.7 ± 3.8	27.2 ± 1.8^AB^	27.3 ± 1.3^AB^	27.4 ± 2.2^AB^	27.2 ± 1.7^AB^
CDR	0.0 ± 0.0	1.0 ± 0.0	0.4 ± 0.2^AB^	0.4 ± 0.3^AB^	0.5 ± 0.1^AB^	0.4 ± 0.2^AB^
CDR SOB	0.0 ± 0.0	4.8 ± 1.1	1.5 ± 1.0^AB^	1.8 ± 1.0^AB^	1.5 ± 1.1^AB^	1.5 ± 0.9^AB^
Composite EM	0.0 ± 0.8	-3.5 ± 0.8	-2.1 ± 1.1^AB^	-0.6 ± 0.4^ABC^	-2.3 ± 1.1^AB^	-2.2 ± 1.1^AB^
Composite NM	0.6 ± 1.1	-3.1 ± 1.8	-0.9 ± 0.9^AB^	-0.8 ± 1.0^AB^	-0.1 ± 0.4^ABD^	-1.3 ± 0.8^AB^

AD, Alzheimer's disease; amMCI, amnestic multidomain mild cognitive impairment; asMCI, amnestic single-domain mild cognitive impairment; CDR, Clinical Dementia Rating; EM, episodic memory *z *score; HC, healthy older controls; MCI, mild cognitive impairment; naMCI, nonamnestic mild cognitive impairment; NM, nonmemory *z *score; SOB, sum of boxes. ^A^*P *< 0.05, MCI subtype compared with HC. ^B^*P *< 0.05, MCI subtype compared with AD. ^C^*P *< 0.05, naMCI compared with asMCI and amMCI. ^D^*P *< 0.05, asMCI compared with naMCI and amMCI.

### Neuroimaging results

**Figure 1:**
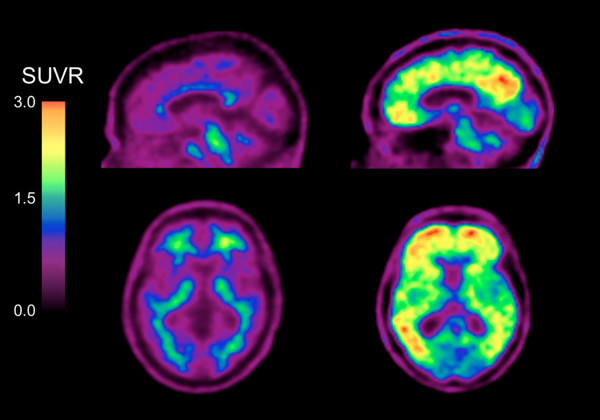
**Sagittal and transaxial ^18^F-florbetaben positron emission tomography images of two mild cognitive impairment participants**. Representative sagittal and transaxial ^18^F-florbetaben positron emission tomography (PET) images of two female mild cognitive impairment participants, both of the same age (73 years old) and with the same Mini-Mental State Examination score of 27. While the PET images on the left show nonspecific retention in white matter, the PET images on the right show high cortical ^18^F-florbetaben retention in the typical pattern seen in Alzheimer's disease, with highest retention in the precuneus/posterior cingulate, frontal and lateral temporal cortices. SUVR, standard uptake value ratio.

Table 2 summarizes the neuroimaging results. Fifty-three percent of MCI participants presented with high Aβ burden as measured by FBB. In the asMCI cases this prevalence rose to 83%, significantly higher than other subcategories. HVs were similar between MCI subgroups (Table 2). Figure 1 shows low and high neocortical FBB retention in two amnestic MCI participants of the same age, gender and Mini-Mental State Examination scores.

**Table 2:** Neuroimaging

				MCI subtype		
				
	HC (*n *= 15)	AD (*n *= 15)	All MCI (*n *= 45)	naMCI (*n *= 4)	asMCI (*n *= 12)	amMCI (*n *= 29)
Neocortical SUVR	1.26 ± 0.22	1.96 ± 0.27	1.54 ± 0.27^AB^	1.50 ± 0.35^B^	1.66 ± 0.20^AB^	1.49 ± 0.28^B^
High β-amyloid, *n *(%)	3 (20)	15 (100)	24 (53)^B^	1 (25)^B^	10 (83)^AC^	13 (45)^B^
Hippocampal volume (cm^3^)			7.1 ± 0.9	7.1 ± 0.8	7.0 ± 0.8	7.2 ± 1.0
WMH volume (cm^3^)			10.2 ± 11.0	13.3 ± 15.5	4.8 ± 6.6^C^	12.0 ± 11.4

AD, Alzheimer's disease; amMCI, amnestic multidomain mild cognitive impairment; asMCI, amnestic single-domain mild cognitive impairment; HC, healthy older controls; MCI, mild cognitive impairment; naMCI, nonamnestic mild cognitive impairment; SUVR, standard uptake value ratio; WMH, white matter hyperintensities. ^A^*P *< 0.05, MCI subtype compared with HC. ^B^*P *< 0.05, MCI subtype compared with AD. ^C^*P *< 0.05, asMCI compared with amMCI.

**Figure 2:**
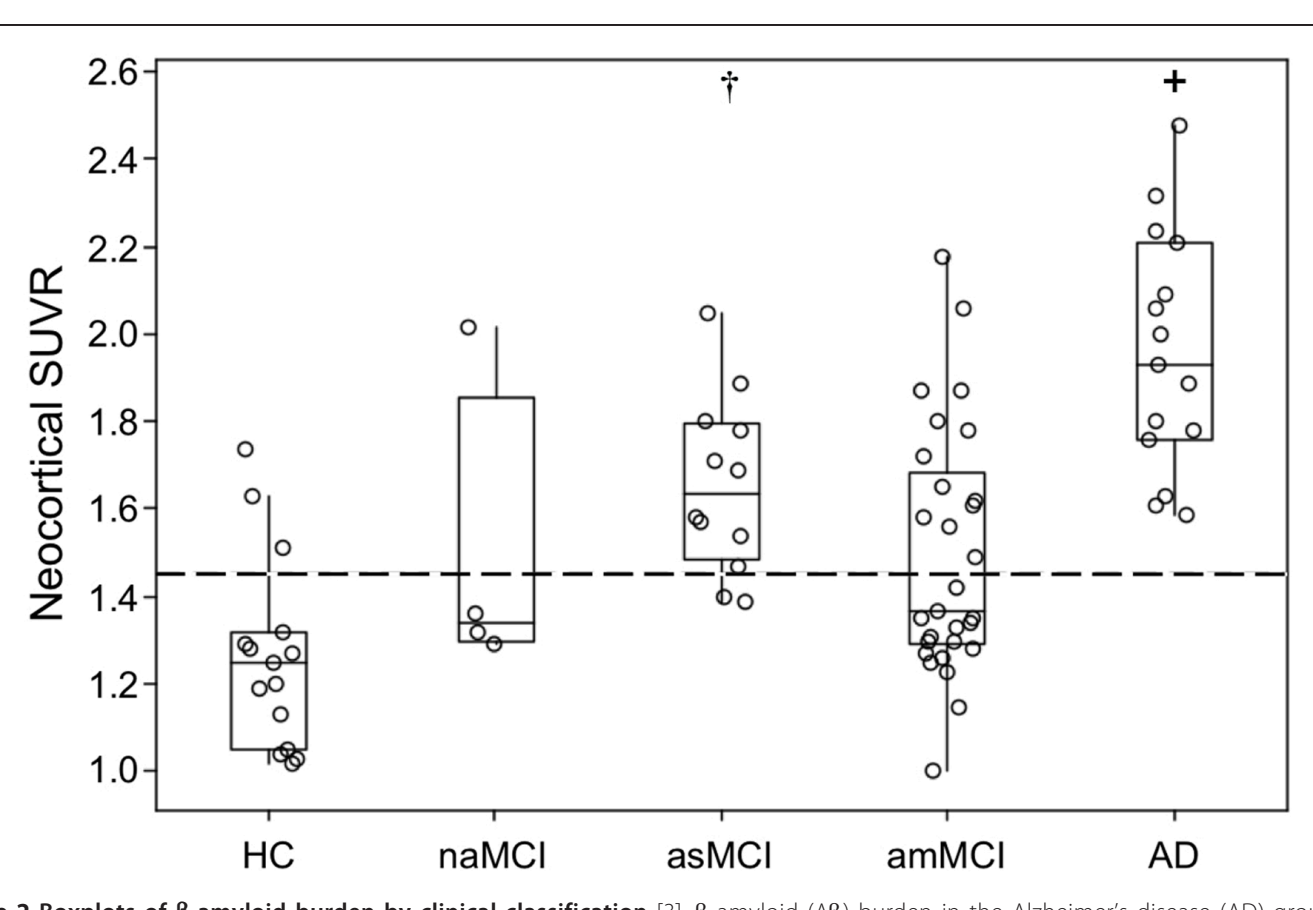
**Boxplots of β-amyloid burden by clinical classification **[3]. β-amyloid (Aβ) burden in the Alzheimer's disease (AD) group was significantly higher (**+**) compared with the mild cognitive impairment (MCI) and healthy control (HC) groups. Aβ burden was high in 83% of the amnestic single-domain MCI (asMCI) participants and significantly higher than in HC (†). Only one nonamnestic MCI (naMCI) subject presented with high ^18^F-florbetaben retention. Dotted line, threshold between high and low Aβ burden. amMCI, amnestic multidomain MCI; SUVR, standard uptake value ratio.

Figure 2 shows the boxplots of neocortical SUVR by clinical subclassification. Only one of the four nonamnestic MCI cases showed high neocortical FBB retention. Ten (83%) asMCI cases had high retention compared with 13 (45%) amMCI cases (Fisher's exact test, *P *= 0.038).

Table 3 shows the characteristics and neuroimaging data in the different MCI subtypes when split into low (SUVR < 1.45) and high (SUVR ≥ 1.45) Aβ groups. MCI participants with high cortical FBB retention performed more poorly on cognitive tasks involving memory. The Clinical Dementia Rating scores were slightly but significantly higher in those with high Aβ deposition. There were no significant differences in HV and WMH between high and low SUVR groups.

**Table 3:** Demographic, cognitive and neuroimaging data in the high and low ^18^F-florbetaben retention groups

	All MCI (*n *= 45)	
	
	Low Aβ (SUVR < 1.45)	High Aβ (SUVR ≥ 1.45)
Number (% of total)	21 (47)	24 (53)
Age	71.8 ± 6.1	73.5 ± 6.9
Years of education	13.5 ± 3.0	13.8 ± 4.2
MMSE	27.9 ± 1.4	26.7 ± 1.9*
CDR	0.4 ± 0.2	0.5 ± 0.0*
CDR SOB	1.3 ± 0.9	1.6 ± 1.0
Composite EM	-1.4 ± 0.8	-2.8 ± 1.0*
Composite NM	-1.0 ± 0.9	-0.8 ± 0.8
Neocortical SUVR	1.30 ± 0.09	1.75 ± 0.19*
Hippocampal volume (cm^3^)	7.4 ± 1.1	6.9 ± 0.8
WMH volume (cm^3^)	9.9 ± 10.2	10.5 ± 11.8

Aβ, β-amyloid; CDR, Clinical Dementia Rating; EM, episodic memory *z *score; MCI, mild cognitive impairment; NM, nonmemory *z *score; SOB, sum of boxes, SUVR, standard uptake value ratio; WMH, white matter hyperintensities. *P < 0.05.

### Correlation analysis

**Figure 3:**
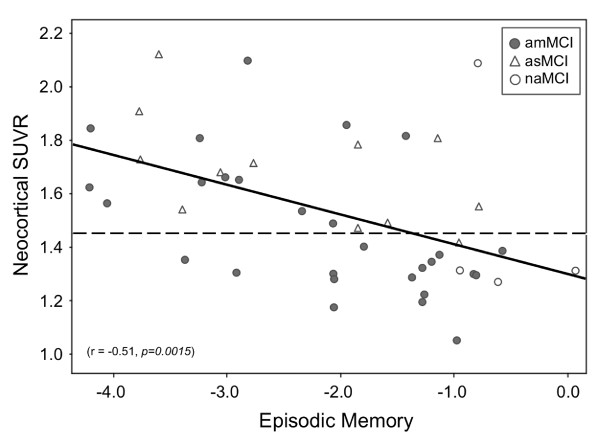
**Relationship between β-amyloid burden and episodic memory in mild cognitive impairment**. There was a significant correlation between β-amyloid (Aβ) burden and memory impairment, which was independent of hippocampal volume. Dotted line, threshold between high and low Aβ burden. amMCI, amnestic multidomain mild cognitive impairment; asMCI, amnestic single-domain mild cognitive impairment; naMCI, nonamnestic mild cognitive impairment; SUVR, standard uptake value ratio.

There was a strong relationship between neocortical SUVR and EM in MCI (*r *= -0.51, *P *= 0.0015) (Figure 3). After accounting for HV, the correlation persisted (*r *= -0.49, *P *= 0.015).

There was also a relationship between HV and EM in the entire MCI cohort (*r *= 0.60, *P *= 0.024). Accounting for neocortical SUVR, the correlation between HV and EM also remained significant (*r *= 0.33, *P *= 0.042) for the entire MCI cohort.

There was a relationship between WMH volume and nonmemory *z *scores (*r *= -0.60, *P *= 0.03; Spearman's ρ = -0.48, *P *= 0.0008). This correlation was amplified in the high SUVR subgroup (*r *= -0.71, *P *= 0.014; Spearman's ρ = -0.57, *P *= 0.0035), but was not present in the low SUVR subgroup (see Figure S1 in Additional file 2). No correlation was found between WMH and neocortical SUVR, HV or EM.

## Discussion

### Aβ burden and memory impairment

This study provides support for the use of FBB PET to assess brain Aβ plaque levels in individuals with MCI. FBB presents with similar characteristics to PiB, including short scan acquisition time and a good safety and tolerability profile. The longer radioactive half-life of fluorine-18 makes FBB PET a promising clinical tool for the detection of AD pathology *in vivo*.

The observation that 53% of scans had high FBB retention is consistent with the prevalence of AD neuropathology at postmortem in those with MCI or in those who progress from MCI to dementia [39,40] and with reports that have used PiB PET or cerebrospinal fluid measures to assess brain Aβ in MCI [41-43]. There was a strong correlation between FBB retention and episodic memory impairment, the cognitive domain that is the best predictor of AD [44]. In contrast to several Aβ imaging studies using PiB [33,45], we found the correlation to be independent of HV - suggesting that Aβ might have a direct effect on memory storage and retrieval. This is supported by functional MRI studies of the default network that have shown a relationship between regional Aβ tracer retention and disrupted synaptic activity well beyond the hippocampus in neuronal memory circuits [46].

Despite the multifaceted nature of memory and other cognitive domains affected by a wide spectrum of physical and environmental factors, the arbitrary distinction of single-domain amnestic MCI from multidomain amnestic MCI appears to increase confidence of *in vivo *AD pathology. In our cohort, approximately 80% of asMCI presented with high FBB retention. The Alzheimer's Disease Neuroimaging Initiative (ADNI) Grand Opportunities criterion for late MCI, which relies on a single test score of logical memory [47], also has value for increasing confidence of *in vivo *Aβ neuropathology [48]. Indeed 24 participants in our cohort could be reclassified as late MCI, and 19 of these (79%) had positive scans. On the contrary, the criterion for early MCI [47] may have value in raising the possibility of neuropathology other than Aβ. In our cohort, eight participants could be reclassified as early MCI using the ADNI Grand Opportunities criteria, and five of these (63%) had negative scans. It would be of interest to know the prognostic value of conversion to AD and non-AD dementia in the different classifications of MCI in our cohort, and longitudinal follow-up of this cohort is ongoing.

The *z *scores were calculated from a demographically matched cohort of participants with normal cognitive scores, normal brain MRI scans and negative PiB scans. Our study therefore included some subjects who did not score 1.5 standard deviations below published means on any of the episodic memory tests, and some subjects who performed poorly on word list recall or complex figure recall but not on the Logical Memory task. Consequently, 13 (29%) participants did not meet ADNI Grand Opportunities criteria for either early or late MCI. This broader definition of MCI may lead to different results. The inclusion of MCI subjects with such a wide range of memory test scores may have allowed a better assessment of the correlation of episodic memory impairment with brain Aβ burden. Significant correlation between Aβ deposition and memory has been reported previously [26,32,33], but has not been consistently found in other studies [43,45]. In contrast to episodic memory, and consistent with previous reports from PiB studies, no association was observed between neocortical FBB retention and composite nonmemory scores [32], supporting the notion that nonmemory domains at the MCI stage are not directly susceptible to Aβ deposition and are more strongly influenced by other neurodegenerative conditions within the MCI cohort.

PiB studies have shown that Aβ deposition is an early event in the development of AD, preceding the clinical phenotype by several years [49]. Furthermore, the accumulation of Aβ is a slow process that tends towards a plateau as dementia progresses [26,50]. The mean neocortical SUVR in the high FBB MCI was 50% higher than in healthy controls with low FBB (1.75 ± 0.19 vs. 1.17 ± 0.11, respectively), and 12% lower than in AD patients (1.75 ± 0.19 vs. 1.96 ± 0.27, respectively). Consequently it can be predicted that Aβ burden in the MCI subjects with high FBB will reach the Aβ burden typical of AD within 5 to 7 years [26,51].

### Hippocampal atrophy

Current hypotheses suggest that memory decline is preceded by hippocampal atrophy, which in turn is preceded by Aβ deposition [33,50]. While Aβ deposition is a hallmark of AD pathology, hippocampal atrophy is a common feature of AD that correlates well with episodic memory dysfunction and has emerged as a biomarker for this condition. However, hippocampal atrophy is not specific for AD and may be found in frontotemporal lobar degeneration, dementia with Lewy bodies and vascular dementia.

In our MCI cohort, accounting for HV had a slight effect on the strong correlation between Aβ burden and EM. After accounting for neocortical SUVR, the correlation between HV and EM was still present but less significant. These results suggest a direct effect of Aβ on memory networks, and are somewhat at odds with the hypothesis that hippocampal atrophy mediates Aβ effects on EM [33]. This discrepancy may be explained by the different approaches in the recruitment of MCI cohorts.

### White matter hyperintensities

Recent work in healthy older and vascular dementia individuals suggested that Aβ deposition and WMH volumes have independent etiologies and independent impacts on cognition [52,53]. While Aβ deposition is associated with altered activity patterns in the default network during memory encoding tasks [46], WMH are associated with a faster decline in global cognitive performance, executive function and processing speed in MCI subjects [54]. This observation is consistent with our finding that the majority (83%) of asMCI in this study had high Aβ deposition and a relatively low WMH volume, where amMCI cases who presented with a more variable FBB retention had significantly higher WMH volumes instead. The higher WMH volumes in the amMCI subtype compared with the asMCI subtype also suggest that cognition in the amMCI subtype is less specifically affected by Aβ deposition compared with the asMCI subtype for it may also be affected by other underlying conditions associated with high WMH volumes [54]. In our MCI cohort there was no direct correlation between WMH volume and Aβ burden. An association between WMH volume and composite scores did present in nonmemory-related tasks but only in the high Aβ burden subjects. This observation supports the notion that there may be a synergistic interaction between Aβ deposition and WMH on nonmemory-related cognitive functions [55], even though no direct relationship between Aβ deposition and nonmemory-related cognitive functions was found.

### Clinical utility of ^18^F-florbetaben PET in MCI

Each of the four MCI subtypes has been proposed to be associated with an increased risk of developing a particular type of dementia [3]. One study showed that while most amnestic MCI progressed to AD, nonamnestic MCI was more likely to progress to other types of dementia [56]. In the current study, 21 (47%) MCI cases had low Aβ burden. Our findings suggest that the cognitive impairment in these MCI participants might not be related to Aβ deposition, and other factors such as depression [57], cerebrovascular disease [54], or non-AD pathologies [10,25] should be considered. A significant proportion of individuals with MCI do not progress to dementia or return to normal [56]. These individuals will probably be in our group with low FBB retention as shown in longitudinal PiB imaging studies of MCI, but longitudinal follow-up of our cohort is required to confirm this hypothesis. In addition to Aβ deposition, environmental factors, brain and/or cognitive reserve and the presence of other age-related diseases may influence and modulate the development and progression of cognitive impairment. To ascertain the clinical utility of Aβ imaging will require follow-up of participants in longitudinal studies. Such studies are underway, including the ADNI and Australian Imaging Biomarkers and Lifestyle (AIBL) trial. Longitudinal follow-up of the present cohort is also in progress.

### Limitations

Limitations of the present study include the relatively small numbers and the single-center setting. Findings from this study warrant validation in a larger multicenter cohort. Moreover, given the wide day-to-day variance of cognitive test scores, longitudinal studies will be needed to further corroborate our initial findings with regards to the association between cognition and neuropathology. Another limitation that might hinder comparison with similar studies is the highly characterized normal cohort used to generate the *z *scores that, with a smaller variance, results in more stringent cutoff values. Our cohort may therefore include participants with minor deficits who would be otherwise classified as normal when published norms standards are applied. On the contrary, all subjects in the study were referred from memory disorder specialists with a clinical diagnosis of MCI, so they represent the patient population likely to be investigated with Aβ imaging. Another limitation of the study is that the brain volumetric assessments pool data obtained on MRI scanners with different field strengths. Given its relevance in memory performance, this study focused on the regional atrophy of the hippocampus, but cortical atrophy in other regions of the brain - such as the posterior cingulate gyrus or the parietal or frontal lobes - might possibly explain some additional variance in memory impairment, thus affecting the observed relationship with Aβ. Further studies assessing regional brain atrophy and its relation to cognition and Aβ are needed to help elucidate the potential interplay between these different factors.

## Conclusion

Higher Aβ deposition in MCI as measured by FBB is associated with more severe memory impairment and is independently correlated with episodic memory impairment after adjusting for hippocampal volume. Moreover, the use of FBB may prove useful in the early differential diagnosis of MCI, identifying subjects with and without brain Aβ, potentially aiding early therapeutic interventions as well as helping to predict prognosis.

## Abbreviations

Aβ: β-amyloid; AD: Alzheimer's disease; ADNI: Alzheimer's Disease Neuroimaging Initiative; amMCI: amnestic multidomain mild cognitive impairment; asMCI: amnestic single-domain mild cognitive impairment; EM: episodic memory *z *scores; FBB: ^18^F-florbetaben; HV: hippocampal volume; MCI: mild cognitive impairment; MRI: magnetic resonance imaging; PET: positron emission tomography; PiB: ^11^C-Pittsburgh Compound B; RCFT: Rey Complex Figure Test; SUVR: standard uptake value ratio; WMH: white matter hyperintensities.

## Competing interests

VLV and CCR were consultants for Bayer Schering Pharma. RSM received research support from Bayer Schering Pharma. CBR, BP and KR are Bayer Schering Pharma employees. The remaining authors declare that they have no competing interests.

## Authors' contributions

KO contributed to participant recruitment, patient screening, data collection, data analysis, and wrote the paper. VLV and CCR contributed to data analysis, and provided comments and critical revision to the paper. AB-F and FL performed neuropsychology assessments and provided comments to the paper. GC provided comments to the paper. PR performed white matter hyperintensity analyses, the method of which was standardized by OS. CBR and BP developed the protocol for clinical trial designs involving FBB. KR performed the statistical analyses. RSM produced FBB in-house. VLV, CLM and CCR are the principal investigators of this study who contributed to trial design. All authors read and approved the manuscript for publication

## Supplementary Material

Additional file 1 **Table S1 presenting exclusion criteria**. Click here for file

Additional file 2 **Figure S1 showing the relationship between WMH and nonmemory scores in MCI subjects with low and high Aβ**. There was a significant correlation between WMH and nonmemory scores in MCI subjects with high Aβ in the brain, but the association was not present in the low Aβ subgroup. naMCI, nonamnestic mild cognitive impairment. Click here for file
