# Supplementary material for: 18F-florbetaben Aβ imaging in mild cognitive impairment
Source: Alzheimers Res Ther. 2013 Jan 16;5(1):4. doi: 10.1186/alzrt158 (PMC3580329; doi:10.1186/alzrt158)
Supplement: Additional file 1 — Table S1 presenting exclusion criteria. [file alzrt158-S1.DOC]

**Supplemenmtary Table 1. Exclusion criteria**

1. **Lifetime history of affective disorder.**
2. **Schizophrenia or schizo-affective disorder.**
3. **Current alcohol or drug abuse.**
4. **Haematological pathology.**
5. **Medications which are judged by the referring clinician to significantly impact cognition, e.g. significant doses of sedatives or acetylcholinesterase inhibitor use.**
6. **History of cancer within 5 years.**
7. **Physical disabilities that would restrict prolonged supine positioning.**
8. **History of relevant neurological disease.**
9. **History of severe head trauma, brain surgery or ICH lesion**
10. **Previous research related radiation exposure resulting in exceeding regulatory limits.**
11. **History of severe anaphylactic reaction.**
12. **Any unstable medical condition.**
13. **Any clinically significant haematological or biochemical results outside of normal range.**
