# Supplementary figures and images for: 18F-florbetaben Aβ imaging in mild cognitive impairment
Source: Alzheimers Res Ther. 2013 Jan 16;5(1):4. doi: 10.1186/alzrt158 (PMC3580329; doi:10.1186/alzrt158)

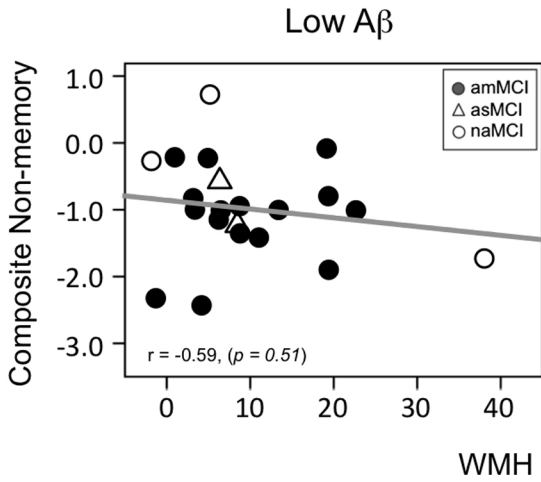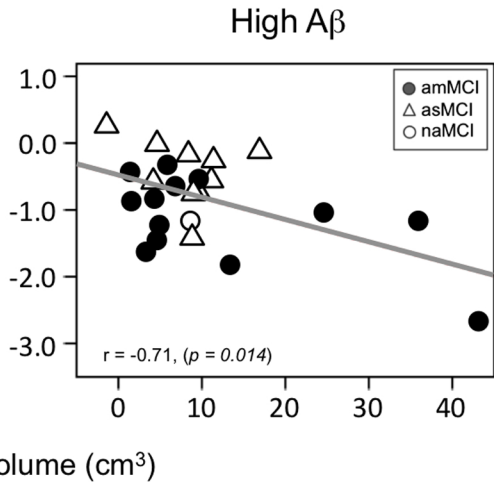

Supplement: Additional file 2 — Figure S1 showing the relationship between WMH and nonmemory scores in MCI subjects with low and high Aβ. There was a significant correlation between WMH and nonmemory scores in MCI subjects with high Aβ in the brain, but the association was not present in the low Aβ subgroup. naMCI, nonamnestic mild cognitive impairment. [file alzrt158-S2.PDF]
